# Hyponatremia and hospital outcomes among patients with pneumonia: a retrospective cohort study

**DOI:** 10.1186/1471-2466-8-16

**Published:** 2008-08-18

**Authors:** Marya D Zilberberg, Alex Exuzides, James Spalding, Aimee Foreman, Alison Graves Jones, Chris Colby, Andrew F Shorr

**Affiliations:** 1 Evi *Med *Research Group, LLC, Goshen, Massachusetts, USA; 2 ICON Clinical Research, San Francisco, California, USA; 3 Astellas Pharma US, Inc., Deerfield, Illinois, USA; 4 Dept. of Pulmonary and Critical Care Medicine, Washington Hospital Center, Washington, District of Columbia, USA

## Abstract

**Background:**

Community-acquired (CAP) and nosocomial pneumonias contribute substantially to morbidity and hospital resource utilization. Hyponatremia, occurring in >1/4 of patients with CAP, is associated with greater disease severity and worsened outcomes.

**Methods:**

To explore how hyponatremia is associated with outcomes in hospitalized patients with pneumonia, we analyzed a large administrative database with laboratory component from January 2004 to December 2005. Hyponatremia was defined as at least two [Na^+^] < 135 mEq/L within 24 hours of admission value.

**Results:**

Of 7,965 patients with pneumonia, 649 (8.1%) with hyponatremia were older (72.4 ± 15.7 vs. 68.0 ± 22.0, p < 0.01), had a higher mean Deyo-Charlson Comorbidity Index Score (1.7 ± 1.7 vs. 1.6 ± 1.6, p = 0.02), and higher rates of ICU (10.0% vs. 6.3%, p < 0.001) and MV (3.9% vs. 2.3%, p = 0.01) in the first 48 hours of hospitalization than patients with normal sodium. Hyponatremia was associated with an increased ICU (6.3 ± 5.6 vs. 5.3 ± 5.1 days, p = 0.07) and hospital lengths of stay (LOS, 7.6 ± 5.3 vs. 7.0 ± 5.2 days, p < 0.001) and a trend toward increased hospital mortality (5.4% vs. 4.0%, p = 0.1). After adjusting for confounders, hyponatremia was associated with an increased risk of ICU (OR 1.58, 95% CI 1.20–2.08), MV (OR 1.75 95% CI 1.13–2.69), and hospital death (OR 1.3, 95% CI 0.90–1.87) and with increases of 0.8 day to ICU and 0.3 day to hospital LOS, and over $1,300 to total hospital costs.

**Conclusion:**

Hyponatremia is common among hospitalized patients with pneumonia and is associated with worsened clinical and economic outcomes. Studies in this large population are needed to explore whether prompt correction of [Na^+^] may impact these outcomes.

## Background

Community-acquired (CAP) and nosocomial pneumonias represent a substantial burden to the US healthcare system. CAP alone is responsible for over 1 million hospital admissions annually [1] and $4.4 billion in hospitalization costs [2]. In addition to CAP, there are nearly 130,000 cases of non-ICU hospital-acquired pneumonia (HAP) and over 100,000 cases of ICU-acquired pneumonia annually [3], the incremental hospital costs of which have been reported to range from $5,800 to $20,000 per case [4-6]. Because of pneumonia's staggering clinical and economic burden, it is important to identify and fully understand all modifiable factors that influence its hospital course.

Hyponatremia is the most common electrolyte imbalance seen in clinical practice [7]. It also frequently accompanies pulmonary diseases, both infectious and neoplastic [8]. In mixed patient populations, others have documented that hyponatremia adversely affects clinical outcomes [9]. With respect to pneumonia, a recent single-center cohort study found the incidence of hyponatremia at hospital admission among CAP patients to be 28% [10]. Importantly, the presence of hyponatremia was associated with not only prolongation of hospitalization (HLOS), but also an increase in hospital mortality.

To extend our understanding of the epidemiology and outcomes of hyponatremia at the time of hospitalization among subjects with pneumonia, and to clarify the association of hyponatremia with the measures of hospital resource utilization, such as the need for mechanical ventilation (MV) and the need for and LOS in the ICU, we conducted a large retrospective cohort study. We hypothesized that hyponatremia at admission is associated with an increased risk of hospital death among patients with pneumonia and that this condition additionally adds significantly to HLOS and costs.

## Methods

No human subjects were prospectively enrolled in the study, and therefore an independent Institutional Review Board (Copernicus Group IRB, Research Triangle Park, NC, USA, #ICO1-08-252) granted an exemption and waived the informed consent requirement.

### Data source description

Data were obtained from Solucient's ACTracker^® ^database, a sample of hospitals that contribute general discharge-level information, in addition to specific drug and laboratory information. Approximately 27 hospitals contribute data at any point in time, with 39 unique hospitals represented over the two-year study period from January 2004 through December 2005. The data source is de-identified in compliance with HIPPA regulations.

### Subjects

We identified persons with pneumonia based on the presence of the International Classification of Disease version 9, Clinical Modification (ICD-9-CM) 3-digit disease code for the principal discharge diagnosis of pneumonia (ICD-9-CM code 486) [11]. We required that patients have at least one laboratory value for serum sodium [Na^+^] available during their hospital admission. For those patients who did not meet the laboratory definition for hyponatremia (see below), we further required that s/he have no primary or secondary diagnosis of hyponatremia (ICD-9-CM code 276.1).

### Hyponatremia definition

Hyponatremia discharges were required to have an admission [Na^+^] below 135 mEq/L on the first or second day of admission. To improve specificity for the diagnosis, we defined hyponatremia to be present if there was at least one additional [Na^+^] below 135 mEq/L within 24 hours of the admission value. We did not specifically seek to exclude pseudohyponatremia.

### Outcomes

Hospital mortality served as the primary study outcome; vital status was determined based on the presence of a hospital discharge disposition of "Died". Because it may be related to severity of illness, we secondarily examined the relationship of admission hyponatremia and the need for MV and ICU in the first 48 hours, on LOS in the ICU, as well as on overall HLOS and incremental hospital costs. A patient's MV utilization was identified by an ICD-9-CM procedure code of 96.7 within 48 hours of admission. An ICU stay was identified by a laboratory test or drug utilization within the ICU in the first 48 hours of admission. By counting the distinct number of days on which a patient had a lab test or drug record in the ICU, we derived the ICU LOS. Costs were estimated using claim charges and adjusted to 2005 US dollars using the medical component of the Consumer Price Index. These adjusted charges were then multiplied by the hospital-specific cost-to-charge ratio, estimated from a Medicare Provider Analysis Review (MEDPAR) file, to yield hospital costs in 2005 US dollars.

### Covariates

We examined multiple potential confounders of the outcomes of interest. In all models covariates were age, gender, race, geographic region, teaching status of the hospital, admission source, principal payer, and Deyo-Charlson Comorbidity Index (Deyo-CCI) score [12,13]. The CCI is a score derived from data abstracted from medical records to arrive at a measure of comorbidity burden [12]. The Deyo-CCI is an adaptation of this method applied to administrative datasets using ICD-9 codes to generate a comorbidity score [13]. The hospital cost and LOS models adjusted for the additional covariates of mortality and ICU and MV stay in the first 48 hours, while the ICU LOS model adjusted for mortality and MV stay in the first 48 hours.

### Statistical analyses

All of the covariates were examined in univariate analyses comparing their prevalence among those with and without hyponatremia. We subsequently adjusted our estimates of the impact of hyponatremia on the outcomes of interest by entering them into multivariable regression models. All unadjusted between-group comparisons were performed using the Wilcoxon rank-sum test or Median score test for continuous variables, and the chi-square test for categorical variables. We used generalized linear models (GLM) to estimate the costs and LOS (both hospital and ICU) that were attributable to hyponatremia in this population, adjusted for covariates. Costs were log-transformed due to the skewed distribution of medical expenditures, and the negative binomial distribution was used to model LOS to account for overdispersion. Logistic regression modeling was performed to estimate the excess risk of MV and ICU need, as well as for hospital mortality, conferred by hyponatremia. Covariates were included in these models based on previous evidence of associations with the outcomes or on biologic or clinical plausibility of such an association. Goodness of fit for these models was assessed using the scaled deviance and Pearson's chi-square statistic. For all analyses, statistical significance was reached when a two-tailed p-value was less than 0.05.

All analyses were performed using SAS 9.1.3 (SAS Institute, North Carolina).

## Results

Of the 198,281 patients in the database, 7,965 (4%) had been diagnosed with pneumonia and met the inclusion criteria, and of those 5,916 had complete cost data. Patients with pneumonia were 45% male, 85% Caucasian, had a mean age of 68.4 ± 21.6 years, and a mean Deyo-CCI (13) of 1.6 ± 1.6. Eight percent (n = 649) of the entire pneumonia population had evidence of hyponatremia.

The baseline characteristics by hyponatremia status are shown in Table 1. Patients with hyponatremia were more likely to be older, and had a greater burden of comorbid illness as signified by a higher Deyo-CCI. There were no gender or racial differences between the groups. Patients with pneumonia who also had hyponatremia were more likely to be at a teaching hospital than at a non-teaching facility.

**Table 1:** Baseline characteristics of pneumonia patients by hyponatremia status

	Hyponatremia present (n = 649)	Hyponatremia absent (n = 7,316)
Mean (SD) age (years)*	72.4 (15.7)	68.0 (22.0)
Proportion male (%)	48.2	44.9
Proportion Caucasian (%)	87.8	85.0
Mean (SD) Deyo-CCI*	1.7 (1.7)	1.6 (1.6)
Proportion in teaching hospitals^†^	33.3	29.5
Source of admission (%)^†^		
Acute Care Facility	0.2	0.7
ER	77.3	71.8
Physician referral	16.2	17.3
Skilled Nursing Facility	1.4	2.0
Transfer	1.1	1.1
Other	0.5	0.7
Missing/Unknown	3.4	6.4

SD = standard deviation; CCI = Charlson Comorbidity Index; ER = emergency room

*p < 0.05 by Wilcoxon rank-sum test

^†^p < 0.05 by chi-square

Hospital mortality, though low in both groups, was greater among patients with hyponatremia (5.4% vs. 4.0%). This difference, however, only approached statistical significance (p = 0.099) (Table 2). The proportion of patients requiring MV (3.9% vs. 2.3%, p = 0.014) or any ICU admission (10.0% vs. 6.3%, p < 0.001) was significantly higher in the hyponatremic than the normonatremic group. Hyponatremia also was associated with increased ICU LOS, and HLOS. Reflecting this, hospital costs were higher among those with hyponatremia (Table 2). Thus, aggregate median hospital costs exceeded $7,000 in the population with hyponatremia compared to $5,732 in persons with no hyponatremia at admission.

**Table 2:** Hospital outcomes of pneumonia patients by hyponatremia status

	Hyponatremia present (n = 649)	Hyponatremia absent (n = 7,316)	p value*
Hospital mortality (%)	5.4	4.0	0.099
Proportion on MV (%)	3.9	2.3	0.014
Proportion in ICU (%)	10.0	6.3	<0.001
Mean (SD) ICU LOS (days)	6.3 (5.6)	5.3 (5.1)	0.069
Mean (SD) HLOS (days)	7.6 (5.3)	7.0 (5.2)	<0.001
Median (95%CI) hospital costs	$7,086 ($3,765–$14,221)	$5,732 ($2,966–$12,290)	0.001

MV = mechanical ventilation; ICU = intensive care unit; SD = standard deviation; HLOS = hospital length of stay; CI = confidence interval

*P-values obtained using Wilcoxon rank-sum test or Median score test for continuous variables, and chi-square test for categorical variables

**Figure 1:**
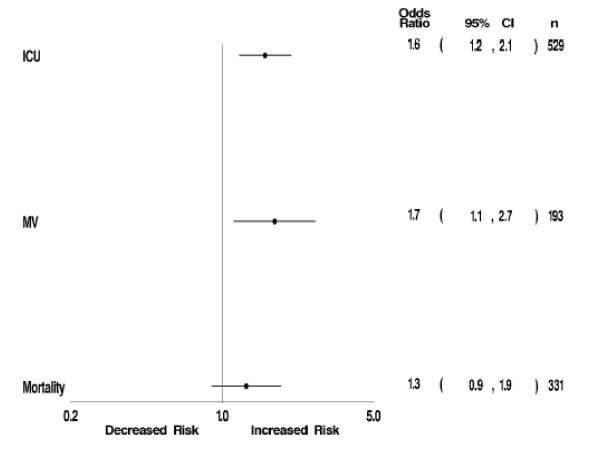
**Adjusted risk for hospital death, need for MV and need for ICU care among hyponatremic compared to normonatremic patients with pneumonia**. Point estimates and 95% confidence intervals, depicting the individual contribution of hyponatremia to the respective outcomes, were derived from logistic regression models utilizing hospital mortality, need for MV and need for ICU as dependent variables, and adjusting for age, gender, race, region, teaching hospital, admission source, principal payer, and Deyo-CCI score.

When the relationship of hospital mortality with hyponatremia was examined in a multivariable logistic regression, adjusting for age, gender, race, geographic region, teaching status of the hospital, admission source, principal payer, and Deyo-CCI score, we observed that hyponatremia was associated with a trend towards an increase in the risk of hospital death (adjusted odds ratio (OR) 1.30, 95% confidence interval (95% CI) 0.90 to 1.87) (Figure 1). The adjusted risk of the need for MV (OR 1.75, 95% CI 1.13 to 2.69) and ICU care (OR 1.58, 95% CI 1.20 to 2.08) within 48 hours of hospital admission were elevated in the group with hyponatremia as well (Figure 1). Hyponatremia was also found to contribute on average 0.8 (95% CI -0.25 to 2.04) and 0.3 (95% CI 0.01 – 0.69) days excess in ICU and HLOS, respectively (Table 3). The marginal hospital costs associated with hyponatremia were $1,324 (95% CI $98 to $2,682) (Table 3). Both the scaled deviance and Pearson's chi-square statistic for these models were close to one, indicating an excellent model fit.

**Table 3:** Incremental impact of hyponatremia on components of hospital resource utilization and costs among patients with pneumonia*

	Incremental increase	95% CI
ICU LOS (days)	0.8	-0.25, 2.04
HLOS (days)	0.3	0.01, 0.69
Hospital costs	$1,324	$98, $2,682

MV = mechanical ventilation; ICU = intensive care unit; LOS = length of stay; HLOS = hospital length of stay *Derived from multivariable linear regression models adjusting for age, gender, race, region, teaching hospital, admission source, principal payer, and Deyo-CCI score, ICU and MV in first 48 hrs, and mortality

## Discussion

We have shown that hyponatremia frequently accompanies hospitalization for pneumonia. Our findings further confirm the independent influence of hyponatremia on hospital length of stay. In addition, we have shown that hyponatremia exerts a negative impact on multiple outcomes such as the need for MV and ICU care, as well as the duration of ICU stay. Financially, hyponatremia adds over $1,300 to the costs of care.

Previous investigations found that hyponatremia in association with a severe Legionella pneumonia requiring an ICU stay is a strong independent predictor of mortality [14]. However, only one prior cohort study from a single institution evaluated the relationship between hyponatremia and outcomes among all patients hospitalized with CAP [10]. In this prospective cohort study, performed between 2002 and 2005, Nair and coworkers found the prevalence of hyponatremia, defined as [Na^+^] < 135 mEq/L in the first hospital-obtained sample, to be 28% among 342 patients enrolled in the study. Although hyponatremia was mostly mild, the investigators found an increase in crude HLOS of 2.3 days and a near tripling of hospital mortality among hyponatremic patients when compared to those without hyponatremia. After adjusting for covariates, the presence of hyponatremia was associated with a 7% (p = 0.03) increase in the risk of hospital death.

Our study, although finding a lower prevalence of hyponatremia, adds to this earlier work. First, by employing a more restrictive definition, we confirm the general impact of hyponatremia on multiple outcomes and help clarify the importance of this factor. We further extend the earlier work in that our data derive from a larger and more generalizable multi-center cohort of patients with pneumonia. Simply put, with a sample size more than ten-fold greater we were able to explore more precisely the impact of hyponatremia on economic outcomes and measures of resource use. The lack of effect of hyponatremia on mortality likely reflects the overall low rate of mortality in our cohort. Reliance on a more stringent definition of hyponatremia may have also contributed to this discordant finding regarding mortality. Since we required a second [Na^+^] measurement, patients with hyponatremia at admission who died prior to having a second [Na^+^] drawn are by definition not included in our mortality analysis. Additionally, to the best of our knowledge, ours is the first study to show an association of hyponatremia with such important components of the hospitalization as the need for MV and the need for and LOS in the ICU, as well as to derive hospital costs attributable to hyponatremia.

Pneumonia is an important driver of healthcare costs. The full burden of hospitalization with pneumonia in the US approaches 1.5 million cases annually and its economic impact may be close to $8 billion [1,2,4-6] in hospital costs alone. Although the majority of patients with CAP are treated in the outpatient setting, hospital-based management is responsible for over 90% of the costs of care for this disease [2]. Several risk stratification algorithms, such as CURB, CURB-65 and Pneumonia Severity Index (PSI), have been developed to help identify patients at high risk of CAP-related complications, and to make appropriate site-of-care decisions [15-17]. The more detailed 20-point PSI containing points for hyponatremia ([Na^+^] < 130 mEq/L) has been found to be better than CURB or CURB-65 at identifying low-risk CAP patients, and thus more helpful at avoiding potentially costly and unnecessary hospitalizations [18]. However, the simplicity of both of the CURB instruments makes them attractive bedside clinical tools. The current study gives rise to the possibility that predictive abilities of the CURB instruments may benefit from the addition of the initial [Na^+^] value without compromising its simplicity.

Along similar lines, and once the decision to admit to the hospital has been made, attention to modifiable determinants of hospital outcomes becomes a critical component of care in patients with pneumonia. As an illustration, a randomized controlled trial showed that a simple intervention consisting of making sure that a CAP patient is sitting out of bed or ambulating for at least 20 minutes during the first 24 hours of hospitalization cut the average HLOS fully by 1 day without an increase in adverse events [19]. Approaches like this demonstrate that identification of and attention to important determinants of outcomes can result in substantial gains in those outcomes. By defining the marginal contribution of hyponatremia to the HLOS and associated costs, our study provides further evidence that hyponatremia needs to be evaluated as a potential target for intervention among hospitalized patients with pneumonia.

Although somewhat novel among patients with pneumonia, hyponatremia has been identified as a predictor of hospital outcomes in other populations. For example, among patients with heart failure there is a well-recognized inverse relationship between admission [Na^+^] and hospital mortality [20,21]. A recent large cohort study of nearly 50,000 patients with acutely decompensated heart failure reported the prevalence of hyponatremia (defined as admission [Na^+^] < 135 mEq/L) of 20% and noted that there is a 20% increase in the risk of hospital death for each 3 mEq/L decrease in [Na^+^] below 140 mEq/L; admission hyponatremia was also independently associated with increased HLOS [22]. Chua et al., in a cohort of 103 geriatric hospitalized patients in the United Kingdom found an 18% prevalence of hyponatremia ([Na^+^] < 135 mEq/L) and a similar association between hyponatremia and outcomes [23]. Contrary to these observed associations, Brouwer and coworkers, while finding a high prevalence of admission hyponatremia (30%), did not uncover any influence on the outcomes among patients hospitalized with community-acquired bacterial meningitis; HLOS, however, was not examined [24]. To sum up, the preponderance of evidence points to a significant association between the presence of hyponatremia at admission and worsened outcomes. The current study furthers this evidence base to the hospitalized population with pneumonia and additionally demonstrates that hyponatremia impacts every component of the aggregate hospital outcomes.

Our study has several important limitations that should be acknowledged. Firstly, because of its observational nature, the crude associations between hyponatremia and outcomes are likely confounded. Notably, we have attempted to address this limitation by performing multivariable analyses. However, the possibility of residual confounding remains. Secondly, by virtue of its retrospective design, the study is prone to several forms of bias. Thirdly, the fact that the data source is not clinical but administrative in nature makes our case definition prone to misclassification. Although the presence of laboratory data and our stringent definition of hyponatremia eliminated that as a limitation of exposure classification, the definition of pneumonia was somewhat less precise, as it relied on the presence of the corresponding ICD-9-CM code. Conversely, our stringent definition of hyponatremia may have itself resulted in an immortal time bias, such that our current estimate of mortality rate among hyponatremic patients is an underestimate of the actual risk of death. That is, by excluding cases without a confirmatory [Na^+^] value we may have eliminated a substantial number of patients with hyponatremia who died prior to having the opportunity to have had the second [Na^+^] checked. As far as the diagnosis of pneumonia, misclassification remains a potential concern, though, if present, it is most likely non-differential leading the estimated associations to appear less strong. Along the same lines, it is also possible that at least some of the included patients were actually misclassified cases of congestive heart failure. Though our dataset precludes us from confirming or refuting its presence, this misclassification is not unusual in clinical practice, and thus should not detract from the relevance of our estimates. Finally, we have not attempted to separate our cohort by the origins of pneumonia (e.g., CAP vs. HAP). Although of importance for future investigations, our aim for the current analysis was to answer a more general question of what role hyponatremia may play in any patient with an infection of the lower respiratory tract, regardless of its etiology.

## Conclusion

In summary, we have shown that hyponatremia is common among hospitalized patients with pneumonia and independently associated with worsened clinical outcomes, as well as with an increase in the utilization of MV, ICU and hospital resources. Future research needs to focus not only on how hyponatremia may affect subpopulations of patients with pneumonia, but also how severity of hyponatremia impacts hospital outcomes. Most importantly, studies are needed to evaluate the role of currently available therapies aimed at correction of hyponatremia in improving the outcomes of patients with pneumonia.

## Competing interests

This project was supported by a grant from Astellas Pharma US, Inc. Drs. Zilberberg and Shorr are consultants to Astellas Pharma US, Inc., who markets an arginine vasopressin antagonist. Drs. Exuzides and Colby, Ms. Foreman and Ms. Graves Jones are employees of ICON Clinical Research, which has received research funding from Astellas Pharma US, Inc. Dr. Spalding is an employee and a stock holder of Astellas Pharma US, Inc. The sponsor contributed to analysis planning but had no veto power over their performance or reporting of the data. All analyses were performed by ICON Clinical Research. No external medical writer was engaged to develop this manuscript.

## Authors' contributions

MDZ participated in the design of the study, data interpretation, drafting and revision of the manuscript for important intellectual content. AE participated in the design of the study, data acquisition and analysis, and drafting of the manuscript. JS participated in the design of the study, data interpretation and revision of the manuscript for important intellectual content. AF participated in the design of the study, data acquisition and analysis, and drafting of the manuscript. AGJ participated in the design of the study, data acquisition and analysis, and drafting of the manuscript. CC participated in data acquisition and analysis, and drafting of the manuscript. AFS participated in the design of the study, data interpretation and revision of the manuscript for important intellectual content.

## Pre-publication history

The pre-publication history for this paper can be accessed here:


